# Prevalence and management of pain in Italian patients with advanced non-small-cell lung cancer

**DOI:** 10.1038/sj.bjc.6601810

**Published:** 2004-05-25

**Authors:** M Di Maio, C Gridelli, C Gallo, L Manzione, L Brancaccio, S Barbera, S F Robbiati, G P Ianniello, F Ferraù, E Piazza, L Frontini, F Rosetti, F Carrozza, A Bearz, M Spatafora, V Adamo, L Isa, R V Iaffaioli, E Di Salvo, F Perrone

**Keywords:** analgesics, cancer pain, lung cancer, Pain Management Index

## Abstract

Pain is a highly distressing symptom for patients with advanced cancer. WHO analgesic ladder is widely accepted as a guideline for its treatment. Our aim was to describe pain prevalence among patients diagnosed with advanced non-small-cell lung cancer (NSCLC), impact of pain on quality of life (QoL) and adequacy of pain management. Data of 1021 Italian patients enrolled in three randomised trials of chemotherapy for NSCLC were pooled. QoL was assessed by EORTC QLQ-C30 and LC-13. Analgesic consumption during the 3 weeks following QoL assessment was recorded. Adequacy of pain management was evaluated by the Pain Management Index (PMI). Some pain was reported by 74% of patients (42% mild, 24% moderate and 7% severe); 50% stated pain was affecting daily activities (30% a little, 16% quite a bit, 3% very much). Bone metastases strongly affected presence of pain. Mean global QoL linearly decreased from 64.9 to 36.4 from patients without pain to those with severe pain (*P*<0.001). According to PMI, 616 out of 752 patients reporting pain (82%) received inadequate analgesic treatment. Bone metastases were associated with improved adequacy and worst pain with reduced adequacy at multivariate analysis. In conclusion, pain is common in patients with advanced NSCLC, significantly affects QoL, and is frequently undertreated. We recommend that: (i) pain self-assessment should be part of oncological clinical practice; (ii) pain control should be a primary goal in clinical practice and in clinical trials; (iii) physicians should receive more training in pain management; (iv) analgesic treatment deserves greater attention in protocols of anticancer treatment.

Pain is a common symptom experienced by cancer patients (Cleary, 2000). Pain prevalence and severity do increase with the extension of disease: about half of all cancer patients report some degree of pain, but this percentage rises to 74% in the advanced and terminal stages (Bonica, 1990). Among cancer patients with advanced disease, pain is moderate to severe in 40–50% and very severe or excruciating in 25–30% (Daut and Cleeland, 1982).

Lung cancer is the first cause of cancer death (Parkin, 2001) and it is mostly diagnosed at an advanced stage. Lung cancer-related pain depends on the location of the primary tumour, its loco-regional extension and metastatic spread. Pain may be secondary to peripheral growth of the primary tumour (e.g. pleural or chest wall involvement) or to nerve involvement (pain in the arm or in the shoulder, or the classic Pancoast syndrome). Sometimes a visceral pain can occur, unrelated to the invasion of local structures, presenting as a nonspecific and vague chest pain referred to the ipsilateral hemithorax (Ginsberg *et al*, 1997). Pain can finally be related to metastatic disease, for example, bone metastases, that occur in approximately one-third of patients (Coleman, 1997), or brain metastases, that can cause headache and symptoms related to intracranial hypertension. Moreover, pain may be unrelated to cancer itself, and rather depend from coexisting diseases (e.g. osteoarthritis), particularly common in elderly patients.

Disease symptoms can have a great impact on functional status and quality of life (QoL). Pain is without any doubt among the most distressing symptoms experienced by cancer patients. A recent study of patients with chronic nonmalignant pain reported significant correlations between severity of pain and QoL (Becker *et al*, 1997). Pain, psychological control and spiritual uncertainty have been indicated as the best predictors of QoL scores in cancer patients (Ferrell *et al*, 1995). In 216 patients with metastatic cancer, Wang *et al* (1999) found that increasing severity of pain was associated with worsening health-related functioning.

Despite the existence of published and well-known guidelines for cancer pain management recommended by the World Health Organization (World Health Organization, 1996), undertreatment of pain remains an outstanding problem for the correct treatment of cancer patients, as already emerged from several studies conducted in different countries (Cleeland *et al*, 1994; Larue *et al*, 1995; Zenz *et al*, 1995; Wang *et al*, 1999). This is disappointing because, with a correct use of WHO analgesic ladder, up to 88% of patients is reported to obtain satisfactory relief from pain (Ventafridda *et al*, 1987; Zech *et al*, 1995). It is recommended that treatment should start at the step of the analgesic ladder appropriate for the severity of pain, and all patients with moderate-to-severe cancer pain, regardless of aetiology, should receive a trial of opioid analgesia (Jacox *et al*, 1994). However, very frequently this does not happen.

Objective of this study is to describe the prevalence of pain among patients affected by advanced non-small-cell lung cancer (NSCLC), the impact of pain on health-related QoL and the adequacy of pharmacologic management of pain.

## PATIENTS AND METHODS

### Patients

All patients with advanced NSCLC enrolled in three multicentre randomised clinical trials of first-line chemotherapy performed by our cooperative group between 1996 and 2001 (The ELVIS Group, 1999; Gridelli *et al*, 2003a, 2003b) were eligible for this study. All three studies were approved by Ethical Committees and all patients gave written informed consent. They had stage IV or IIIB (with supraclavicular metastatic nodes or malignant pleural effusion) disease and a baseline performance status not worse than 2, according to ECOG scale (Oken *et al*, 1982). Patients with clinically overt brain metastases were not eligible.

In the ELVIS study (Elderly Lung cancer Vinorelbine Italian Study) vinorelbine was compared with supportive care alone in patients ⩾70 years (The ELVIS Group, 1999). Vinorelbine was given 30 mg m^−2^ on days 1 and 8 every 3 weeks, for six cycles. The primary end point of the trial was health-related QoL. Recruitment started in April 1996 and overall 191 patients were randomised until November 1997.

The MILES study (Multicentre Italian Lung cancer in the Elderly Study) was conducted in the same age group of the ELVIS trial, and compared the combination of vinorelbine and gemcitabine *vs* the two single drugs (Gridelli *et al*, 2003a). Patients were randomly assigned vinorelbine alone (30 mg m^−2^), gemcitabine alone (1200 mg m^−2^), or combination of vinorelbine (25 mg m^−2^) plus gemcitabine (1000 mg m^−2^). All treatments were delivered on days 1 and 8 every 3 weeks for six cycles. The primary end point was overall survival; 707 patients were randomised between December 1997 and November 2000.

The GEMVIN study was conducted with adult (<70 years) patients, randomly assigned either combination of vinorelbine (25 mg m^−2^, days 1 and 8) plus gemcitabine (1000 mg m^−2^, days 1 and 8) or cisplatin-based chemotherapy: cisplatin (80 mg m^−2^, day 1) plus either gemcitabine (1200 mg m^−2^, days 1 and 8) or vinorelbine (30 mg m^−2^, days 1 and 8) for six cycles of 21 days (Gridelli *et al*, 2003b). The study aimed to assess whether the combination of gemcitabine and vinorelbine improved QoL, without shortening survival, compared to standard platinum-containing regimens. Accrual started in Italy in October 1998, and in Canada in May 1999. Overall, 503 patients (414 in Italy) were randomised between October 1998 and March 2001. In this secondary analysis, only Italian patients are considered.

### Assessment of pain and QoL

The European Organization for Research and Treatment of Cancer (EORTC) core questionnaire (QLQ-C30) and the lung-cancer-specific module (QLQ-LC13), evaluating specific symptoms of lung cancer, were used to evaluate QoL (Aaronson *et al*, 1993; Bergman *et al*, 1994). Both questionnaires are designed to be completed by the patient.

Pain is assessed by two items in the QLQ-C30: item 9 (*During the past week have you had pain*?) and item 19 (*During the past week did pain interfere with your daily activities*?). Further three items addressing pain during the last week are present in the QLQ-LC13: item 10 (*Have you had pain in your chest?*), item 11 (*Have you had pain in your arm or shoulder?*) and item 12 (*Have you had pain in other parts of your body*). All these items are scored in four categories (*Not at all/A little/Quite a bit/Very much*): in this paper, these categories have been renamed as *no pain, mild, moderate and severe pain*, respectively. In the present analysis, in order to describe the relationship between pain and QoL and to evaluate the adequacy of analgesic treatment, the worst pain reported by each patient at baseline assessment (merging item 9 of QLQ-C30 and items 10, 11, 12 of QLQ-LC13) has been considered.

Global QoL is assessed by item 29 (*How would you rate your overall physical condition during the past week*?) and item 30 (*How would you rate your overall QoL during the past week*?) of QLQ-C30. Both items are scored 1 (*Very poor*) to 7 (*Excellent*). Functional scales assessed by the QLQ-C30 are physical functioning (items 1–5), role functioning (items 6 and 7), emotional functioning (item 21–24), cognitive functioning (items 20 and 25) and social functioning (items 26 and 27). According to the Manual of the EORTC QoL Study Group (Fayers *et al*, 1999), global QoL and functioning scales were rescaled by calculating the mean raw scores of single items and transforming them linearly so that each ranged from 0 to 100. For Global QoL and functioning scales, the higher the value the better the level of function.

### Protocol requirements and data collection on analgesic treatment

In all the three trials, study protocols recommended that analgesic treatment should have been prescribed on an *around-the-clock* basis and not only ‘as needed’. World Health Organization three-step analgesic ladder (World Health Organization, 1996) was recommended. This strategy consists of three steps of increasing analgesic potency: (1) a nonopioid (e.g. *a nonsteroidal anti-inflammatory drug or acetaminophen*); (2) a weak opioid for mild to moderate pain (e.g. *tramadol or codeine*); and (3) a strong opioid for moderate-to-severe pain (e.g. *morphine or fentanyl*).

The three trials had the same Case Report Form for data collection on supportive drugs. Data were collected from the starting date of chemotherapy until interruption, for up to seven drugs in each cycle of chemotherapy (corresponding to a theoretic 21-days period), with the daily dose and the number of days of assumption recorded. The CRF had to be filled in by the Investigator after each cycle, so the data refer to the actual assumption by the patient, and not to the intended prescription.

Analgesic drugs assumed by patients have been coded according to the Anatomical Therapeutic Chemical (ATC) classification system (World Health Organization Collaborating Centre for Drug Statistics Methodology, 2002; Di Maio *et al*, 2003) and according to the WHO three-step analgesic ladder as for the analgesic potency.

### Pain management index

In the present analysis, only analgesic drugs assumed during the 3 weeks following baseline assessment (i.e. the time period corresponding to the first cycle of chemotherapy) were considered. Analysis has been limited to 21 days in order to evaluate the adequacy of analgesic treatment in relation to pain assessed at baseline. According to the WHO guidelines, management of cancer pain is considered adequate when there is consistency between the level of pain reported by the patient and the potency of the analgesic drug prescribed.

The Pain Management Index (PMI) (Cleeland *et al*, 1994) compares within each patient the most potent analgesic prescribed with the self-reported level of pain. As in the original paper, for each patient we derived the highest level of analgesic used, out of four possible levels: no analgesic drug (score 0); a nonopioid, for example, a nonsteroidal anti-inflammatory drug or acetaminophen (score 1); a weak opioid, for example, tramadol or codeine (score 2); a strong opioid, for example, morphine (score 3). We then defined the patient's level of pain as the worst pain score reported in the baseline QoL questionnaires (item 9 of QLQ C30 and items 10, 11, 12 of QLQ LC13). The absence of pain was scored as 0, mild pain as 1, moderate pain as 2 and severe pain as 3.

The PMI, computed by subtracting the pain level from the analgesic level, ranges from −3 (a patient suffering severe pain but not receiving analgesic drugs) to +3 (a patient receiving a strong opioid and reporting no pain). Negative scores are considered to indicate inadequate analgesic treatment, and scores of 0 or higher are considered to be a conservative indicator of acceptable treatment.

### Statistical methods

Patients studied had been enrolled in three different randomised clinical trials (RCT), two addressed to older people (⩾70 years) and one limited to adult patients (<70 years). To counteract differences in pain assessment and baseline covariates that could eventually occur among the three trials, all analyses were adjusted by stratifying by RCT. A preliminary comparison of baseline covariates was assessed by χ^2^ test.

Stratified Wilcoxon rank-sum test was used to investigate relationship between the worst pain reported and baseline characteristics (StatXact^©^, CYTEL software Corp. 2002); differences of worst reported pain among RCTs were assessed by Kruskal–Wallis nonparametric ANOVA.

Impact of pain on QoL scales was evaluated by Jonckheere–Terpstra test because of the ordered nature of both variables.

Association between adequacy of pain management and baseline covariates was assessed by Mantel–Haenszel test stratified by RCT. Factors affecting adequate pain management were finally evaluated by a stratified logistic regression model; the explanatory variables of the model, defined ‘*a priori*’, were gender, Performance Status (2 *vs* 0–1), stage (IV *vs* IIIB), bone metastases (yes *vs* no) and worst pain as a continuous variable. In this model, age could not be evaluated because it was completely confounded by stratification. To investigate the role of age a further logistic model not stratified by RCT was fitted, where age (⩾70 *vs* <70) was added to all the previous covariates.

## RESULTS

**Figure 1:**
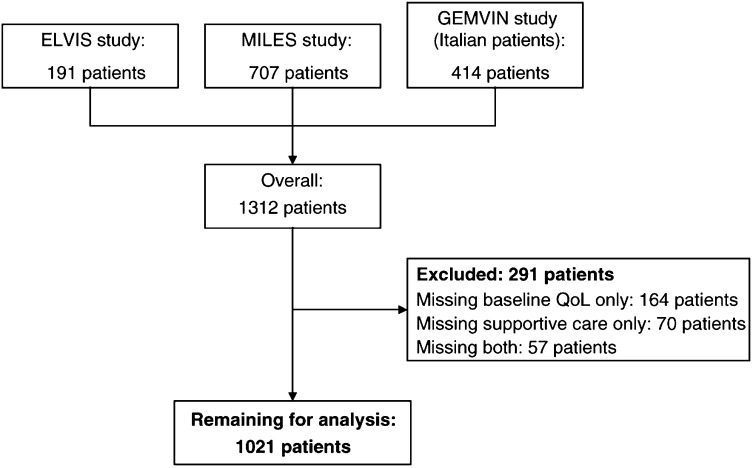
Flow-chart of the study.

Overall, 1312 patients had been enrolled in the three studies. Of these, 291 patients (22.2%) were excluded from this analysis: 164 patients (12.5%) for missing baseline QoL questionnaire, 70 (5.3%) for missing data about supportive drugs and 57 (4.3%) for missing both sets of information. Thus, a database of 1021 cases was used for all the analyses (Figure 1

).

Baseline characteristics are described in Table 1

**Table 1:** Baseline characteristics of the 1021 patients analysed by clinical trial

	**Trial**			
	**ELVIS**	**MILES**	**GEMVIN**	***P*^a^**
Number of patients	135 (13%)	561 (55%)	325 (32%)	
				
*Age, years*				n.a.
Median	74	74	62	
Range	(69^b^ – 85)	(63^b^ – 85)	(35 – 72^c^)	
				
*Gender*				0.43
Male	117 (87%)	462 (82%)	266 (82%)	
Female	18 (13 %)	99 (18%)	59 (18%)	
				
*Performance status*				0.03
0–1	103 (76%)	461 (82%)	281 (86%)	
2	32 (24%)	100 (18%)	44 (14%)	
				
*Stage*				0.01
IIIB	40 (30%)	176 (31%)	72 (22%)	
IV	95 (70%)	385 (69%)	253 (78%)	
				
*Bone metastases*				<0.001
Absent	116 (86%)	449 (80%)	231 (71%)	
Present	19 (14%)	112 (20%)	94 (29%)	

a χ^2^ test; n.a. = not applicable because trials were different by design (see text).

b Seven patients (one ELVIS, six MILES) randomised although <70 years.

c Two patients randomised although >70 years.

. Overall median age was 72 years (range 35–86) and was strongly affected by 696 patients (68%) older than 70 enrolled in the ELVIS and MILES trials. Most of the patients were males (83%), had metastatic disease (72%, with bone metastases in 22%) and a good Performance Status (0 or 1 in 83%). Limited differences were observed among trials, but for bone metastases, more frequent in adult subjects.

### Prevalence of pain

Overall, pain was reported by 752 patients (74%, 95% CI 71–77%); it was mild in 42%, moderate in 24% and severe in 7% (Table 2

**Table 2:** Pain reported by the 1021 patients at the baseline QoL assessment

	**Number of patients (%)**				
	**No answer**	**Not at all**	**A little**	**Quite a bit**	**Very much**
*EORTC QLQ C30:*					
During the past week:					
(a) Have you had pain?	11 (1)	392 (38)	370 (36)	206 (20)	42 (4)
(b) Did pain interfere with your daily activities?	7 (1)	507 (50)	307 (30)	166 (16)	34 (3)
					
*EORTC QLQ LC13:*					
(c) Have you had pain in your chest?	10 (1)	664 (65)	267 (26)	72 (7)	8 (1)
(d) Have you had pain in your arm or shoulder?	8 (1)	563 (55)	303 (30)	119 (12)	28 (3)
(e) Have you had pain in other parts of your body?	14 (1)	640 (63)	246 (24)	97 (9)	24 (2)
					
Worst pain reported (a/c/d/e)	—	269 (26)	433 (42)	245 (24)	74 (7)

). Chest pain was reported by 34% of patients and was moderate or severe in 8%; pain in arm or shoulder was reported by 44% and scored moderate or severe in 15%. Table 3

**Table 3:** Relationship between worst pain and main baseline characteristics (*n*=1021)

	**Number of patients (%)**				
	**Not at all**	**A little**	**Quite a bit**	**Very much**	
	**269 (26)**	**433 (42)**	**245 (24)**	**74 (7)**	
*Clinical trial*					***<0.001***^a^
ELVIS	26 (19)	67 (50)	35 (26)	7 (5)	
MILES	171 (30)	238 (42)	123 (22)	29 (5)	
GEMVIN	72 (22)	128 (39)	87 (27)	38 (12)	
					
*Age*					***<0.001***^a^
<70 years	73 (22)	129 (39)	90 (27)	38 (12)	
⩾70 years	196 (28)	304 (44)	155 (22)	36 (5)	
					
*Gender*					*0.69*^b^
Male	221 (26)	363 (43)	205 (24)	56 (7)	
Female	48 (27)	70 (40)	40 (23)	18 (10)	
					
*Performance status*					***<0.001***^b^
0–1	241 (29)	364 (43)	187 (22)	53 (6)	
2	28 (16)	69 (39)	58 (33)	21 (12)	
					
*Stage*					***<0.001***^b^
IIIB	89 (31)	136 (47)	50 (17)	13 (5)	
IV	180 (25)	297 (41)	195 (27)	61 (8)	
					
*Bone metastases*					***<0.001***^b^
Absent	249 (31)	347 (44)	164 (21)	36 (4)	
Present	20 (9)	86 (38)	81 (36)	38 (17)	

a Kruskal–Wallis test.

b Wilcoxon rank-sum test stratified by clinical trial.

shows the relationship among worst pain and baseline characteristics of the patients: moderate/severe pain was more frequent in patients younger than 70 (39 *vs* 27%), in patients with worse performance status (45 *vs* 28%), in patients with metastatic disease (35 *vs* 22%) and in those with bone metastases (53 *vs* 25%). No significant association was evident between pain and gender.

### Impact of pain on QoL

As shown in Table, 507 patients (50%) stated that their pain was affecting daily activities (a little in 30%, quite a bit in 16% and very much in 3%). Relationship among baseline pain and functional scales of health-related QoL are detailed in Table 4

**Table 4:** Mean scores of baseline global health status and functioning scales by worst pain categories

	**Worst pain reported**				
	**Not at all**	**A little**	**Quite a bit**	**Very much**	***P***^a^
Global health status	64.9	56.9	45.4	36.4	<0.001
Physical functioning	88.3	82.4	74.2	68.2	<0.001
Role functioning	79.4	71.3	58.4	54.7	<0.001
Emotional functioning	78.4	70.7	62.3	53.2	<0.001
Cognitive functioning	90.9	85.9	78.2	77.0	<0.001
Social functioning	85.2	81.4	72.7	69.8	<0.001

a Jonckheere–Terpstra test.

. All QoL scales were strongly affected by pain. Mean global QoL score linearly decreased from 64.9 to 36.4 from patients with no pain to those with severe pain (*P*<0.001). Mean scores of the functioning scales (physical, role, emotional, cognitive and social) also linearly decreased with increasing pain severity.

### Adequacy of analgesic treatment

Analgesic medications assumed by the patients during the first 3 weeks following the baseline assessment are reported in Table 5

**Table 5:** Analgesic drugs assumed by the 1021 patients during the first 3 weeks

**ATC categories**	***n*° (%) of patients**
*M01A anti-inflammatory products, nonsteroids*	*140 (14%)*
M01AB acetic acid derivatives (e.g.diclofenac, ketorolac)	107 (10%)
M01AC oxicam (e.g. piroxicam)	4 (<1%)
M01AE propionic acid derivatives (e.g. ibuprofen, naproxen)	15 (1%)
M01AX other anti-inflammatory, nonsteroids (e.g. nimesulide)	18 (2%)
	
*N02 analgesics*	*167 (16%)*
*N02A opioids*	*72 (7%)*
N02AA morphine	26 (3%)
N02AB fentanyl	7 (1%)
N02AC dextropropoxyphene	1 (<1%)
N02AE buprenorphine	7 (1%)
N02AX tramadol	32 (3%)
	
*N02B other analgesics and antipyretics*	*97 (9%)*
N02BA salicylic acid and derivatives	3 (<1%)
N02BE anilides (e.g. acetaminophen)	23 (2%)
	
*N02BE in association with opioids* (codeine+acetaminophen)	*71 (7%)*

(grouped as ATC categories) and Table 6

**Table 6:** Pain management during the 3 weeks following baseline assessment

	**Worst pain reported at baseline assessment (number of patients (percentage by column))**				
	**Not at all**	**A little**	**Quite a bit**	**Very much**	**Total**
*Type of analgesic assumed*					
No analgesics	254 (94)	**344 (79)**	**130 (53)**	**37 (50)**	765 (75)
WHO 1	15 (6)	73 (17)	**77 (31)**	**19 (26)**	184 (18)
WHO 2	—	12 (3)	18 (7)	**9 (12)**	39 (4)
WHO 3	—	4 (1)	20 (8)	9 (12)	33 (3)

Values in bold indicate inadequate pain management. Pain management is considered adequate with a Pain Management Index ⩾0 (Cleeland *et al*, 1994).

(grouped as WHO three-step ladder). Despite 74% of the patients referred some pain at baseline, only 25% assumed analgesics. Notably, no analgesic treatment was assumed by 79% of patients with mild pain, 53% of those with moderate pain and 50% of those suffering from severe pain.

Drugs falling in the first step of the WHO analgesic ladder were the most frequently used: 184 out of 256 patients receiving analgesics (72%) and 169 out of 752 patients reporting pain (22%). Patients with moderate or severe pain received an opioid in 18% of cases (a weak opioid in 8% and a strong opioid in 9%). Strong opioids, mostly orally administered slow-release morphine (fast-release preparations were not widespread in Italy when the patients analysed in this study were treated), were assumed by 4.4% of patients reporting pain (33 out of 752), in particular by 1, 8 and 12% of patients reporting mild, moderate and severe pain, respectively.

In all, 82% of patients (616 out of 752 with any pain) had negative scores of the Pain Management Index, indicating inadequate analgesia (Table 6). The inadequacy rate tended to increase with increasing pain severity: 79, 84 and 88% among patients reporting mild, moderate and severe pain, respectively, possibly as effect of more stringent criteria for adequacy with increasing pain severity. Relationship between adequacy of pain management during the first 3 weeks and main baseline characteristics of the patients are detailed in Table 7

**Table 7:** Relationship between adequacy of pain management during the first 3 weeks and main baseline characteristics (*n*=752 patients reporting pain)

	**Number of patients (%)**		
	**Adequate pain management**	**Inadequate pain management**	***P***
*Clinical trial*			**0.005**^a^
ELVIS	31 (28)	78 (72)	
MILES	69 (18)	321 (82)	
GEMVIN	36 (14)	217 (86)	
			
*Age*			**0.02**^a^
<70 years	35 (14)	222 (86)	
⩾70 years	101 (20)	394 (80)	
			
*Gender*			0.45^b^
Male	116 (19)	508 (81)	
Female	20 (16)	108 (84)	
			
*Performance status*			0.26^b^
0–1	103 (17)	501 (83)	
2	33 (22)	115 (78)	
			
*Stage*			0.09^b^
IIIB	29 (15)	170 (85)	
IV	107 (19)	446 (81)	
			
*Bone metastases*			**<0.001**^b^
Absent	79 (14)	468 (86)	
Present	57 (28)	148 (72)	

a χ^2^ test.

b Mantel–Haenszel test.

. A higher percentage of adequately treated patients was observed among subjects older than 70 as compared to the younger ones (20 *vs* 14%, *P*=0.02). Adequate pain treatment was more frequent in patients with bone metastases than in those without (28 *vs* 14%, *P*<0.001). No significant association with adequacy of pain treatment was evident for gender, performance status and stage of disease. At binary logistic regression, stratified by clinical trial, the presence of bone metastases (OR 2.7, 95% CI 1.7–4.2, *P*<0.0001) and worst reported pain (OR 0.6, 95% CI 0.4–0.8, *P*=0.003) were independent predictors of the adequacy of the analgesic treatment (Table 8

**Table 8:** Predictors of adequacy of analgesic treatment. Binary logistic model stratified by clinical trial

**Covariate**	**Odds ratio^a^ (95% confidence intervals)**	***P***
Gender	0.92 (0.54 – 1.56)	0.75
Performance status	1.35 (0.85 – 2.14)	0.20
Stage	1.05 (0.64 – 1.72)	0.86
Bone metastases	2.70 (1.73 – 4.21)	<0.0001
Worst pain reported	0.62 (0.45 – 0.85)	0.003

a A higher odds ratio is associated with a greater likelihood of adequate treatment of pain.

). When the unstratified logistic model was fitted with age added as explanatory variable, older subjects were found to be slightly associated with more adequate analgesic therapy (OR 0.6, 95% CI 0.4–1.0, *P*=0.05).

## DISCUSSION

This study provides a description of the occurrence and the severity of pain in a large series of patients with advanced NSCLC. In addition, it provides two major findings, that is, a close relationship between pain and QoL indicators and a clear evidence of inadequate analgesic treatment in a large proportion of the patients.

Knowledge of the prevalence of pain at diagnosis in patients affected with advanced cancers, for whom palliation is frequently the primary goal of treatment, may help planning organisation of therapeutic units and strategy of clinical research could be better planned if reasonable estimates of the major patients' needs were available. To the best of our knowledge this is the largest series of patients with advanced NSCLC in which prevalence of pain at diagnosis is described. This was possible by pooling the data of three prospective randomised clinical trials of chemotherapy, performed by the same cooperative group and coordinated by the same centre (The ELVIS Group, 1999; Gridelli *et al*, 2003a, 2003b). All these trials had very similar selection criteria (except for age that was discriminant among the studies), study procedures (all the patients filled-in EORTC questionnaires at baseline) and recommendations about supportive care. The pooled database should be sufficiently representative of the patients' population with advanced NSCLC who are candidate to a systemic treatment with chemotherapy (Perrone *et al*, 2002). Because in all of these trials patients with performance status worse than 2 according to ECOG (Oken *et al*, 1982) were excluded, our study does not address patients with more deteriorated health status, that are probably those more frequently suffering pain. Such information could only derive by clinical trials or observational studies specifically dedicated to terminally ill patients. We found that three-fourths of patients suffer some degree of pain and in about one-third of cases this pain does significantly affect daily activities. These figures are consistent with a smaller series of patients with various stages of lung cancer (61 cases with localised disease, 91 with regional, 68 metastatic and 40 with undetermined extension), in which pain was assessed by a graphic rating scale combining visual, numerical and descriptive indicators: only 71 out of 256 patients (27.7%) stated they had had no pain in the last week, while 50.7% had suffered moderate to very bad pain (Greenwald *et al*, 1987). Overall, these data confirm that pain is an issue of great relevance in the clinical presentation of advanced NSCLC. In the data we are reporting, the presence of bone metastases seems to be the key variable associated with occurrence and severity of pain; indeed, the associations of clinical trial, age and stage with pain (Table 3) can overall be explained with a slightly higher prevalence of bone metastases in the GEMVIN study, among patients younger than 70, and, obviously, in stage IV disease.

An important finding of the present study is that pain has a strong impact on the QoL of cancer patients. In most functioning scales, indeed, a clinically relevant change of 6–10 points (Osoba *et al*, 1998) was observed with increasing severity of pain. Although this could be expected, based on other studies with similar findings in smaller series of patients (Ferrell *et al*, 1995; Becker *et al*, 1997; Wang *et al*, 1999), it is impressing that the mean score of self-assessed global QoL for patients without pain is almost twice as much as for patients with severe pain, and a progressive and clinically significant (Osoba *et al*, 1998) decrease in mean scores is seen for patients with mild and moderate degree of pain. QoL is currently considered as a primary end point of treatment and clinical trials planning (American Society of Clinical Oncology, 1996), and global scores (e.g. that coming from items 29–30 in the EORTC C-30 QLQ) are usually considered as primary outcome measures. The present data suggest that, in cases where a high prevalence of pain is expected at baseline, pain could be as sensitive as primary outcome measure as QoL global score.

Based on such considerations, one could argue that adequate pain relief could significantly improve overall QoL. Even, the improvement of QoL provided by adequate analgesic treatment could probably exceed that attributable to the most effective anticancer treatments, particularly for a disease, like advanced NSCLC, not highly sensitive to specific treatments. Unfortunately, in the present study, we found that more than 80% of patients suffering some degree of pain received inadequate analgesic medications, based on WHO ladder strategy for pain management and the definition of adequacy proposed by Cleeland *et al* (1994) with the Pain Management Index. This Index is obviously a simplification, because it does not take into account the dose but only the type of analgesic drugs, and it does not consider the assumption of the so-called ‘adjuvant’ analgesics (corticosteroids, antidepressants, anticonvulsants) that might play an important role in cancer pain management. However, the same Index has been repeatedly used when similar surveys have been realized in different countries (Cleeland *et al*, 1994, 1997; Larue *et al*, 1995; Wang *et al*, 1999; Beck and Falkson, 2001; Yun *et al*, 2003). We found that, at multivariate analysis, pain treatment adequacy significantly improves in presence of bone metastases, showing that the knowledge of bone metastases stimulates a proper consideration of pain treatment by physicians. On the other side, the fact that adequacy of treatment is inversely correlated with severity of pain suggests that the level of communication between patients and physicians regarding pain is frequently inadequate. This suggests that the use of self-filled pain scales could be effective in clinical practice to draw physicians'attention on pain treatment also in the subgroup of patients without bone metastases. Experimental data show that the use of self-filled QoL questionnaires in clinical practice improves the effectiveness of patient–physician communication (Detmar *et al*, 2002; Velikova *et al*, 2004).

Several factors can produce undertreatment of pain. On the patient's side, reluctance to report pain (e.g. because of concerns about distracting physicians from treatment of underlying disease or fear that pain means worse disease) has been considered (Cleary, 2000). Furthermore, patients often refuse treatment with opioid drugs for unjustified fear of addiction. On the physician's side, in addition to failures in pain assessment (Von Roenn *et al*, 1993), inadequate pain management can occur. This might either lie on cultural pitfalls or concerns regarding the use of analgesic, for example, possible side effects, risk of patients addiction and problems due to strict regulations for the use of controlled substances (morphine and other strong opioids). The latter issue has probably played a pivotal role in many countries, including Italy. Indeed, the absence of consistent national policies on palliative care and the legal restrictions on the use and availability of opioid analgesics has been pointed out by an expert committee of WHO (World Health Organization, 1996). Considering morphine consumption as a surrogate index of pain management, Italy was at the lowest levels of consumption in Europe, in a monitoring action performed by WHO (World Health Organization, 2000). The data of the present study confirm a trend toward an inappropriately low use of opioids. However, a new law on the medical use of analgesics has been approved in Italy in February 2001 (Gazzetta Ufficiale della Repubblica Italiana, 2001), when virtually all the patients analysed here had already been treated. This law significantly simplifies the procedures for the prescription of opioids; the possible increase of opioids consumption could eventually translate into a more adequate pain management, although latest reports are not encouraging (Mercadante, 2002).

A final note should be done as for age of patients, because older age has been indicated as a risk factor for pain undertreatment (Cleeland *et al*, 1994; Cleeland, 1998; Bernabei *et al*, 1998). A possible explanation is that toxic effects like gastrointestinal bleeding with NSAID and constipation or cognitive failure with opioids can be particularly ill-tolerated in elderly patients. If this were true, because two of the three clinical trials pooled in this study were dedicated to patients older than 70, adequacy of pain treatment could have been underestimated in the present analysis. Because the trials we analysed were dedicated to different patient populations selected on the basis of the age limit at 70 years, we cannot adequately test the age effect on pain treatment in multivariate analysis stratified by clinical trial. However, within an unstratified model that could be confounded by trial, we found that undertreatment was even more frequent in the younger than in the older patients, thus not confirming conclusions from previous publications.

In conclusion, some recommendations can be done based on our data: (i) tools for self-assessment of pain should be used in clinical practice management of patients with advanced cancer, to reduce underestimation of the symptom; (ii) pain should be clearly identified as preminent within more generic QoL definition and pain control should be directly addressed as a primary goal of treatment both in clinical practice and in clinical trials; (iii) training of physicians in the correct management of pain should be encouraged at all levels, to improve the appropriate use of analgesic drugs; (iv) great attention has to be paid to analgesic treatment in protocols of anticancer treatment, to avoid frustrating possible specific effects because of inadequate supportive care.

## Appendix

### List of Participating Investigators and Institutions

National Cancer Institute: Clinical Trials Unit (Francesco Perrone, Massimo Di Maio, Ermelinda De Maio, Enrico Di Salvo), Medical Oncology B^*^ (Cesare Gridelli^1^, Antonio Rossi^1^, Emiddio Barletta, Maria Luisa Barzelloni^2^, Paolo Maione^1^, Rosario Vincenzo Iaffaioli), Naples; Medical Statistics, Second University, Naples (Ciro Gallo, Giuseppe Signoriello); Medical Oncology, S Carlo Hospital, Potenza^*^ (Luigi Manzione, Domenico Bilancia, Angelo Dinota, Gerardo Rosati, Domenico Germano); Monaldi Hospital: Pneumology V (Francovito Piantedosi, Alfredo Lamberti, Vittorio Pontillo, Luigi Brancaccio, Carlo Crispino), Oncology (Alfonso Illiano, Maria Esposito, Ciro Battiloro, Giovanni Tufano), Naples; Mariano Santo Hospital: Pneumology (Santi Barbera, Francesco Renda, Francesco Romano, Antonio Volpintesta), Medical Oncology, Cosenza; Oncologic Day-Hospital, Civil Hospital, Rovereto (Sergio Federico Robbiati, Mirella Sannicolò); Medical Oncology, Rummo Hospital, Benevento (Giovanni Pietro Ianniello, Vincenza Tinessa, Maria Grazia Caprio); University Federico II, III Internal Medicine, Naples^*^ (Silvio Cigolari^3^, Angela Cioffi, Vincenzo Guardasole, Valentina Angelini, Giovanna Guidetti); Oncology, Sacco Hospital, Milan (Elena Piazza, Virginio Filipazzi, Gabriella Esani, Anna Gambaro, Sabrina Ferrario); Medical Oncology, S Paolo Hospital, Milan (Luciano Frontini^4^, Sabrina Zonato, Mary Cabiddu^5^, Alberto Raina^6^); Medical Oncology, San Lazzaro Hospital, Alba (Federico Castiglione, Gianfranco Porcile, Oliviero Ostellino); Medical Oncology, ULSS 13, Noale (Francesco Rosetti, Orazio Vinante, Giuseppe Azzarello); Oncology, La Maddalena Hospital, Palermo^*^ (Vittorio Gebbia, Nicola Borsellino, Antonio Testa); Medical Oncology, Civil Hospital, Legnano (Sergio Fava, Anna Calcagno, Emanuela Grimi); Oncology, Cardarelli Hospital, Campobasso (Sante Romito, Francesco Carrozza); Medical Oncology, S Maria della Misericordia Hospital, Udine (Cosimo Sacco, Angela Sibau); Medical Oncology, San Gennaro Hospital, Naples (Luigi Maiorino, Antonio Santoro, Massimiliano Santoro); Medical Oncology, S Luigi and SS Currò Gonzaga Hospital, Catania^*^ (Giuseppe Failla, Rosa Anna Aiello); Medical Oncology, CRO, Aviano (Alessandra Bearz, Roberto Sorio, Simona Scalone); Medical Oncology, Civil Hospital, Padova (Silvio Monfardini, Adolfo Favaretto, Micaela Stefani); Pneumology, University, Palermo (Mario Spatafora, Vincenzo Bellia, Maria Raffaella Hopps); Ospedali Riuniti: Pneumology (Giovanni Michetti, Maria Ori Belometti); Pneumooncology, Forlanini Hospital, Roma (Filippo De Marinis, Maria Rita Migliorino, Olga Martelli); Medical Oncology, University, Messina (Vincenzo Adamo, Giuseppe Altavilla, Antonino Scimone); Medical Oncology, S Maria Goretti Hospital, Latina^*^ (Enzo Veltri, Modesto D’Aprile, Giorgio Pistillucci); Pneumology, S Luigi Gonzaga Hospital, Orbassano (Giorgio Scagliotti, Silvia Novello, Giovanni Selvaggi); Medical Oncology, Molinette Hospital, Turin (Oscar Bertetto, Libero Ciuffreda, Giuseppe Parello); Medical Oncology, University, Perugia (Maurizio Tonato, Samir Darwish); Medical Oncology, USSL 33, Rho (Giuliana Mara Corradini, Gianfranco Pavia); Oncology, Serbelloni Hospital, Gorgonzola (Luciano Isa, Paola Candido); Oncology, Civil Hospital, Polla (Nestore Rossi, Antonio Calandriello); Medical Oncology, S Giuseppe Hospital, Milan (Maurizia Clerici, Roberto Bollina, Paolo Belloni); Medical Oncology, S Vincenzo Hospital, Taormina (Francesco Ferraù, Emilia Malaponte); Oncologic Radiotherapy, S Gerardo Hospital, Monza (Antonio Ardizzoia); Thoracic Surgery, University, Foggia (Matteo Antonio Capuano, Michele Angiolillo, Nicolino D’Aloia); Medical Oncology, Civil Hospital, Avellino (Mario Belli, Giuseppe Colantuoni); Medical Oncology, Az. Ospedaliera ‘Bianchi-Melacrino-Morelli’, Reggio Calabria (Giampietro Gasparini^7^, Alessandro Morabito^7^, Domenico Gattuso^7^); Experimental Medical Oncology, Oncologic Institute, Bari (Giuseppe Colucci, Domenico Galetta, Francesco Giotta); Oncology, Fatebenefratelli Hospital, Benevento^*^ (Tonino Pedicini, Antonio Febbraro, Cesira Zollo); Oncology—Hematology, C.Poma Hospital, Mantova (Franca Pari, Enrico Aitini); Oncology, S Maria Hospital, Terni (Francesco Di Costanzo^8^, Roberta Bartolucci, Silvia Gasperoni^8^); Medical Oncology, ULSS 15, Camposampiero (Fernando Gaion, Giovanni Palazzolo); Oncology, Miulli Hospital, Acquaviva delle Fonti^*^ (Giuseppe Nettis, Annamaria Anselmo); Medical Oncology, Civil Hospital, Treviglio (Sandro Barni, Marina Cazzaniga); Medical Oncology, S Carlo Borromeo Hospital, Milan (Gino Luporini, Maria Cristina Locatelli); Medical Oncology, S Giovanni Hospital, Turin (Cesare Bumma, Alfredo Celano, Sergio Bretti^9^); Medical Oncology, S Chiara Hospital, Trento (Enzo Galligioni, Orazio Caffo), Trento; Thoracic Surgery, Ascalesi Hospital, Naples; Oncology, Cottolengo Hospital, Turin; Medical Oncology, University, Milano; Medical Oncology, University La Sapienza, Rome; Medical Oncology, S Francesco di Paola Hospital, Paola^*^; Medical Oncology, University, Sassari; Regina Elena Institute: Medical Oncology, Medical Oncology II^*^, Rome; Medical Oncology, S Croce Hospital, Fano; Chemotherapy, University, Palermo^*^; Pneumology, S Martino Hospital, Genova; Medical Oncology, S Andrea Hospital, Vercelli; Medical Oncology, USL 5-Ovest Vicentino; Medical Oncology, S Bortolo Hospital, Vicenza; Oncology, CROB, Rionero in Vulture; Medical Oncology, Bergamo; Medical Oncology, Centro Catanese di Oncologia, Catania^*^; Oncology, Agnelli Hospital, Pinerolo; Pneumology, S Corona Hospital, Garbagnate; Medical Oncology, G Di Maria Hospital, Avola; Oncohematology (Medicine I), Maggiore Hospital, Lodi; Medical Oncology, Hospital, Lecco; Oncology, Civil Hospital, Gorizia; Oncology, S Paolo Hospital, Bari; Medical Oncology, Fondazione Salvatore Maugeri, Pavia; Medical Oncology, Biomedical Campus, Rome; Tisiology and Pneumology, Second University, Monaldi Hospital, Naples; Medical Oncology, ASL Lodi, Casalpusterlengo; Medicine, Civil Hospital, Lagonegro; Oncology, Civil Hospital, Sciacca^*^; Medical Oncology, University, Businco Hospital, Cagliari; Medical Oncology, University, Cagliari; Pneumology, Policlinico S Matteo, Pavia; Oncology, S Giovanni di Dio e Ruggi d’Aragona Hospital, Salerno^*^; Medical Oncology, USL 1, Sassari; Medical Oncology, Civil Hospital, Legnago; Medical Oncology I, IST, Genova; Medical Oncology, Regional Hospital, Bolzano; Businco Oncologic Hospital, Cagliari; Pneumology, Circolo Varese Hospital, Varese; Oncology, Civil Hospital, Ariano Irpino; Oncology, SS Trinità Hospital, Sora; Pneumology, Galateo Hospital, S Cesario di Lecce^*^; Medical Oncology, Maggiore Hospital, Trieste; Medicine, Civil Hospital, Vigevano; Medical Oncology, Casa di Cura Igea, Milan; Oncohematology, Pugliese Ciaccio Hospital, Catanzaro; da Procida Hospital: Pneumology, Salerno; Geriatric Oncology, Civil Hospital, S Felice a Cancello; Oncology, C Cantù Hospital, Abbiategrasso; Pneumology, Crema Hospital, Crema; Medical Oncology, Civil Hospital S Maria delle Grazie, Pozzuoli.

^*^Members of GOIM (Gruppo Oncologico Italia Meridionale).

Present addresses: ^1^S Giuseppe Moscati Hospital, Avellino; ^2^da Procida Hospital, Salerno; ^3^S Giovanni di Dio e Ruggi d’Aragona Hospital, Salerno; ^4^S Gerardo Hospital, Monza; ^5^Azienda Ospedaliera di Treviglio-Caravaggio, Treviglio; ^6^S Pio X, Milan; ^7^S Filippo Neri Hospital, Rome; ^8^Careggi Hospital, Florence; ^9^Civil Hospital, Ivrea.
